# Reproductive biology of male common dolphins (*Delphinus delphis*) in New Zealand waters

**DOI:** 10.1007/s00227-023-04266-5

**Published:** 2023-10-06

**Authors:** Emily I. Palmer, Emma L. Betty, Sinéad Murphy, Matthew R. Perrott, Adam N. H. Smith, Karen A. Stockin

**Affiliations:** 1 Cetacean Ecology Research Group, School of Natural Sciences, Massey University, 0745 Auckland, New Zealand; 2 Marine and Freshwater Research Centre, Department of Natural Resources & the Environment, School of Science and Computing, Atlantic Technological University, ATU Galway City, Old Dublin Road, Galway, H91 T8NW Ireland; 3 School of Veterinary Sciences, Massey University, Palmerston North, New Zealand; 4 School of Mathematical and Computational Sciences, Massey University, 0745 Auckland, New Zealand

**Keywords:** Life history, Reproduction, Density dependence, Fisheries interactions, Management, SDG14

## Abstract

Reproductive parameters were assessed in 64 male common dolphins (*Delphinus delphis*) examined post-mortem from strandings and bycatch in New Zealand between 1999 and 2020. The stages of male sexual maturation were assessed using morphological measurements and histological examination of testicular tissue. Age was determined via growth layer groups (GLGs) in teeth. The average age (ASM) and length (LSM) at attainment of sexual maturity were estimated to be 8.8 years and 198.3 cm, respectively. Individual variation in ASM (7.5–10 years) and LSM (190–220 cm) was observed in New Zealand common dolphins. However, on average, sexual maturity was attained at a similar length but at a marginally younger age (< 1 year) in New Zealand compared to populations in the Northern Hemisphere. All testicular variables proved better predictors of sexual maturity compared to demographic variables (age and total body length), with combined testes weight the best outright predictor of sexual maturity. Reproductive seasonality was observed in male common dolphins, with a significant increase in combined testes weight in austral summer. This aligns with most other studied populations, where seasonality in reproduction is typically observed. Given the known anthropogenic impacts on New Zealand common dolphins, we recommend that these findings be used as a baseline from which to monitor population-level changes as part of conservation management efforts.

**Supplementary Information:**

The online version contains supplementary material available at 10.1007/s00227-023-04266-5.

## Introduction

An understanding of male reproductive parameters can provide insights into population structure, intraspecific competition, mating behaviours and systems, group structure, and sexual dimorphism within a population or species (delBarco-Trillo and Ferkin 2004; Dixson and Anderson 2004; Bronson 2009; De Bruyn et al. 2011). Within Cetacea (whales, dolphins, and porpoises), a wide range of reproductive parameters are displayed, including large variation in average age and length at attainment of sexual maturity and gonadal size (Clapham 1996; Ramos et al. 2000; Danilewicz et al. 2004; MacLeod and MacLeod 2009; Nicolosi and Loy 2019).

Spermatogenesis, the production of haploid spermatozoa, is the primary indicator of sexual development in male cetaceans (Plön and Bernard 2007; Katsumata 2010; Kemper et al. 2014). Reproduction is predominantly seasonal in cetaceans, with most males producing sperm only at certain times of the year (Sørensen and Kinze 1994; Neimanis et al. 2000; Vu et al. 2015; Robeck and O'Brien 2018). Sexual features of male cetaceans vary widely, but may include a prominent post-anal hump, large testes size, long penises, and retained bodily scarring caused by intraspecific interactions (Neumann et al. 2002; Dixson and Anderson 2004; Dines et al. 2014, 2015; Betty et al. 2019). In life-history studies, sexual maturity is typically assessed via the gross and histological examination of testes post-mortem (Reddy 1996; Murphy et al. 2005; Kesselring et al. 2019), although hormone analysis of blubber, plasma, and blow exhalant samples are increasingly used for live, free-ranging individuals (Hogg et al. 2009; Kellar et al. 2009; Galligan et al. 2018; Robeck et al. 2019).

Reproductive parameters of male cetaceans have been assessed across many species (Olesiuk et al. 2005; Matkin et al. 2014; Wells 2014; Betty et al. 2019; Plön et al. 2020), with variation often linked to body size and longevity. For example, male harbour porpoises (*Phocoena phocoena*) in Scottish waters attain sexual maturity at 5 years of age and at 132 cm in length (Learmonth et al. 2014); whereas male bottlenose dolphins (*Tursiops aduncus*) in the Indo-Pacific attain sexual maturity at c. 12 years of age and between 208 and 220 cm in length (Kemper et al. 2014). Furthermore, there is considerable variation between populations and ecotypes of the same species (Olesiuk et al. 1990; Robeck and Monfort 2006; Chen et al. 2011). For example, male harbour porpoises in the Celtic and Irish seas obtained sexual maturity at a significantly larger body length than those in the North Sea (Murphy et al. 2020). Additionally, individuals in this region experience mixing with the larger, recently proposed sub-species of Iberian harbour porpoises, which would also increase the length at sexual maturity shown by males (Murphy et al. 2020).

The reproductive biology of male common dolphins (*Delphinus delphis*) has been examined in several Northern Hemisphere populations including the western (Westgate and Read 2007) and eastern (Murphy et al. 2005) North Atlantic, the eastern tropical Pacific (Oliver 1973) and the North Pacific (Ferrero and Walker 1995). Less is known for common dolphins in the Southern Hemisphere, with only a single study in the western South Atlantic (Grandi et al. 2022). Such paucity of data has been of concern for Australasia, where common dolphins are subject to several anthropogenic impacts including pollutants (Lavery et al. 2008; Stockin et al. 2021a, b), tourism (Neumann and Orams 2005, 2006; Stockin et al. 2008; Meissner et al. 2015), and fisheries bycatch (Hamer et al. 2008; Stockin et al. 2009; Thompson et al. 2013; Allen et al. 2014; Mackay and Goldsworthy 2017; Barceló et al. 2021; Parra et al. 2021).

As part of the wider Australasian population, New Zealand common dolphins demonstrate significant genetic connectivity with their Australian counterparts (Barceló et al. 2021), which raises concerns about the sustainability of levels of fisheries bycatch on both sides of the Tasman Sea (Du Fresne et al. 2007; Stockin et al. 2009; Thompson et al. 2013; Allen et al. 2014; Mackay and Goldsworthy 2017). However, management units are currently based on Australia and New Zealand having separate populations (Barceló et al. 2021). Given recent declines of common dolphins observed internationally (e.g., in the Mediterranean Sea; Bearzi et al. 2003; Piroddi et al. 2011; Vella et al. 2021), careful management based on a fuller understanding of the reproductive biology of male common dolphins on both sides of the Tasman Sea is required to ensure the long-term viability of the species in this region. As male life history is comparatively less described (Chivers 2009), this study will complement recently published data on female common dolphin reproductive parameters (Palmer et al. 2022), offering population-level insights to common dolphins in New Zealand waters.

Here, we assessed the reproductive biology of male common dolphins in New Zealand waters using histological examination of testicular tissue and a set of testicular measures (combined testes length, combined testes weight, an index of testicular development, and mean seminiferous tubule diameter). Specifically, these testicular parameters were used to examine (1) how testis characteristics change with age, body length, and sexual maturity, (2) the average age and length at attainment of sexual maturity, (3) potential indicators of sexual maturity, and (4) evidence of reproductive seasonality in mature males.

## Materials and methods

### Sample collection

**Fig. 1:**
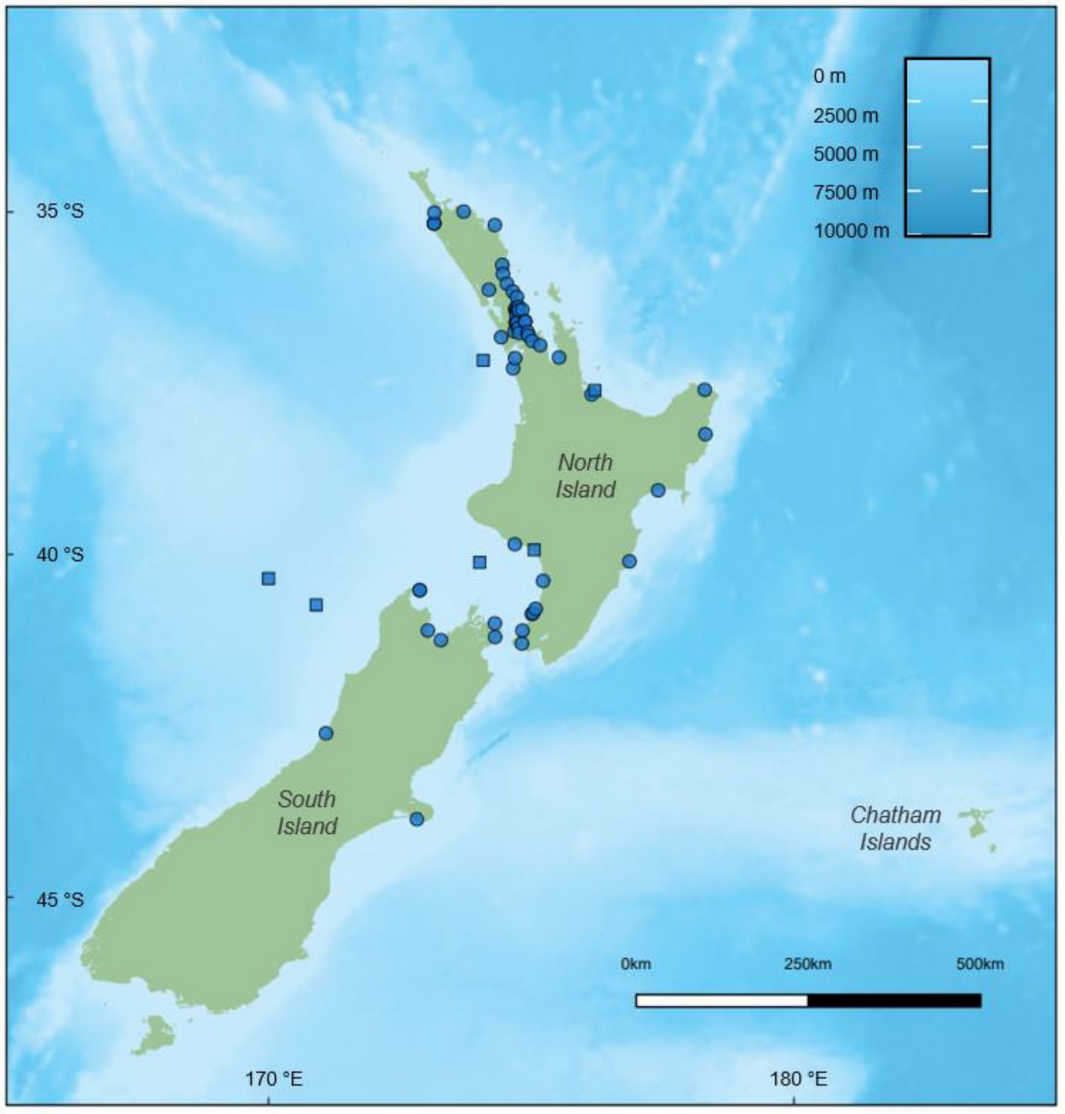
Location of male common dolphin (*Delphinus delphis*) stranding (blue circles) and bycatch (blue squares) events around New Zealand, from which male reproductive samples were collected for this study (*n* = 62)

Reproductive data were collected and assessed post-mortem in 64 male common dolphins following Geraci and Lounsbury (2005). The sample size included 56 individuals (54 independent events) that either live-stranded or were found beachcast on the New Zealand coastline between 1999 and 2020. Additionally, six individuals incidentally captured within the commercial fishery for jack mackerel (*Trachurus novaezelandiae*) off the west coast of the North Island between 2001 and 2003 were included (Fig. 1). The origin of a further 2 males remain unknown.

Testes and associated epididymides were removed and the testes weighed without the associated epididymis to the nearest 0.1 g. Measurements of the length of each testis were taken to the nearest 0.1 cm. Small samples (approx. 1 cm^3^) were subsequently dissected from each testis and epididymis and were fixed in 10% neutral buffered formalin. Total body length (TBL) was measured to the nearest 0.5 cm. Teeth were carefully extracted for age determination following methods outlined in Murphy et al. (2014). Decomposition state was further noted for each individual (*fresh*, *mild*, and *moderate*) as per Stockin et al. (2007).

### Age estimation

Age was estimated by examining decalcified, stained thin sections of tooth from an individual (Murphy et al. 2014). A binocular microscope (10–40 × magnification) was used to examine sections and age was estimated by counting the annual growth layer groups (GLGs) in the dentine as described by Myrick Jr et al. (1983) and Lockyer (1995). Tooth sections were initially read blind (i.e., with no prior biological information known), by at least two of three experienced readers (SM, EB, and EP). Best age estimates or age ranges were subsequently compared, and in the case of any disagreement, further teeth were sectioned and examined again until a final estimate was determined (after Westgate and Read 2007). Individuals that could not be aged reliably (i.e., due to tooth damage or wear) were excluded from further analysis. A neonate was identified if the neonatal line was not present or just forming in the dentine of the tooth.

### Histological assessment of reproductive organs

Stages of sexual maturity were determined by histological examination of testicular tissue (Murphy et al. 2005; Betty et al. 2019). Testicular tissue was processed using standard histological techniques, i.e., by dehydration, clearing, and embedding in paraffin wax. Tissues were sectioned at 5 µm, stained with H&E (haematoxylin and eosin), and mounted on glass slides. Histological slides were examined microscopically (100–400x), and the stage of sexual maturity was determined via assessment of all seminiferous tubules in approximately 1 cm^2^ section of the testicular tissue. Parameters used to assess maturity included the mean diameter of seminiferous tubules (mean DT), the relative proportion of Sertoli cells, interstitial tissue, germinal cells (spermatogonia, spermatocytes, spermatids, and spermatozoa), the activity in the epididymis, and the presence and proportion of spermatozoa in the epididymis (Murphy 2004; Murphy et al. 2005). Neimanis (1996) suggested that in slightly autolysed tissue, the basement membrane may detach from the seminiferous epithelium in some areas, but this would not significantly change the diameter measurements of the tubules in comparison to fresh tissue. Therefore, a mean diameter of the seminiferous tubules was taken by measuring the basement membranes of ten tubule cross-sections. These measurements were collected from images taken using Axiocam 105 with associated Zeiss software (Zeiss 2013) and ImageJ, an image analysis system (Schneider et al. 2012). Only clear circular tubules were measured to ensure an accurate cross-section of the longitudinal axis were taken, in accordance with other cetacean reproductive studies (Neimanis et al. 2000; O’Hara et al. 2002; Betty et al. 2019). If the tissue was observed to be in a state of moderate or advanced autolysis, measurements were not taken as the basement membrane could not be clearly distinguished.

Males were classified into three maturity stages; immature, pubescent, and mature (Murphy et al. 2005; Kemper et al. 2014). Maturity stages were based on the presence and proportion of cell types in the seminiferous tubules, as follows: immature individuals had only Sertoli cells and spermatogonia present; pubescent individuals had both spermatogonia and spermatocytes present; mature individuals had all stages of spermatogenesis present in the tubules (including spermatids and spermatozoa).

### Statistical analysis

#### Models of maturity status given length, age, and testicular measurements

A dataset of six individual-level variables was compiled for 35 males, including two demographic variables (age and TBL) and four testicular variables (combined testes length, combined testes weight, index of testicular development, and mean seminiferous tubule diameter). The index of testicular development was calculated as the combined testes weight in grams (excluding epididymis) divided by the combined testes length in millimetres (Hohn et al. 1985). Relationships among these six variables were explored using charts and Spearman’s rank correlation coefficients. The index variable was log-transformed as it was found to be a better predictor of sexual maturity than its raw form.

The average age (ASM) and length (LSM) at attainment of sexual maturity were estimated for male common dolphins using two methods: (1) Bayesian modelling as detailed hereafter and (2) the sum-of-fraction of immature (SOFI) method (Hohn 1989, see S1 in Supplementary Material).

Progression through the maturity stages (immature, pubescent, and mature) was modelled with Bayesian cumulative logit models fitted with the ‘brms’ package for R (Bürkner 2017). To compare the utility of each of the individual-level variables to predict maturity stage, each variable was used as the single predictor variable (*x*) in turn. Maturity stage (*Y*) was treated as an ordinal variable with three categories (immature, pubescent, and mature) represented as *k* = {1, 2, 3}, respectively. The probability of a male being in stage *k* or below (π_*k*_ = *P*(*Y* ≤ *k*)) was modelled as

$$\mathrm{log}\left(\frac{{\pi }_{k}}{{1- \pi }_{k}}\right)= {\alpha }_{k}- \beta {x}_{i}$$

for *k* = 1, 2; π_3_ = 1 − π_2_. For all three parameters (*α*_1_, *α*_2_, and *β*), weakly informative prior distributions [Student′ s *t*(3, 0, 10)] were assumed. To estimate *x*_50_, the value of *x* at which 50% of males were classified as mature, the posterior distribution of *α*_2_⁄*β* was used. The posterior distribution was then summarised using the mean and 95% credible intervals (‘CrI’; based on 2.5% and 97.5% percentiles).

#### Comparison of models

The relative utility of the demographic and testicular variables as indicators of maturity in the cumulative logic models was compared using the Bayesian LOO (Leave-One-Out) estimate of the expected log pointwise predictive density (using the ‘elpd_loo’ package for R; Vehtari et al. (2017)). ELPD-LOO is a criterion used to estimate out-of-sample predictive accuracy—that is, how accurately a model will predict new data that were not used in the fitting of the model (Vehtari et al. 2017). ELPD-LOO scores are used to compare models fit to the same dataset (Vehtari et al. 2017). In our case, not all individuals had data for all six variables available (due to tissue quality) and so only 35 complete cases were available for comparing all the fitted models with ELPD-LOO. There was only one case of a ‘pubescent’ male in the ‘complete’ dataset, so this case was excluded from the comparison of the models. Thus, a dataset of 34 complete cases was used for comparisons and the pubescent category was omitted. For the more specific comparison of the models with age and TBL as predictor variables, we used a larger data set (*n* = 52) with all the complete cases for these two predictor variables. Two models were fitted to each of the four testicular variables (combined testes weight, combined testes length, index of testicular development, and mean diameter of the seminiferous tubules) as these variables had skewed distributions. One of the models used the raw values (*x*) and the other used the log-transformed values (log *x*). These models were compared based on the ELPD-LOO criterion, which indicated that the log-transformed variables were a better fit. Therefore, we present the models that use the log-transformed testicular variables.

#### Quantifying reproductive seasonality

To assess potential reproductive seasonality in mature males, the variation in mean diameter of seminiferous tubules and combined testes weight between seasons, and across the year (using Julian dates) was tested using Kruskal–Wallis and randomisation tests. Due to the small sample size, males were grouped into austral seasons instead of months following Murphy et al. (2005) and Westgate and Read (2007). Austral seasons were defined as summer (December–February), autumn (March–May), winter (June–August), and spring (September–November). Differences between combined testes weight and mean seminiferous tubule diameter among seasons were assessed with t tests. The testicular and seasonality data were first tested for normality using Shapiro–Wilk tests.

All statistical analyses were conducted using R version 2021.4.1.1 (R Development Core Team 2021).

## Results

### Stages of sexual maturation

Of the 64 males assessed, 37 (58%) and 24 (38%) were classified as immature and mature, respectively. A further 3 (5%) were classified as pubescent (Table 1). Of the 59 males that had decomposition state recorded, 18 (31%) were classified as fresh, 27 (46%) as mild, and 14 (23%) as moderate.

**Table 1:** Mean (± SE), range, and number of samples obtained for each variable [TBL, age, combined testes weight, combined testes length, an index of testicular development (index), and seminiferous tubule diameter] at each stage of male sexual maturation (immature, pubescent, and mature) for common dolphins (*Delphinus delphis*) examined in the study (1999–2020)

Stages	*n*	TBL (cm)	Age (years)	Combined testes weight (g)	Combined testes length (mm)	Log_index (g/mm)	Seminiferous tubule diameter (μm)
Immature	37	145 (± 6)	2.9 (± 0.4)	15.3 (± 2.6)	143.5 (± 8.0)	0.1 (± 0.0)	17.3 (± 0.9)
		89–200	0–9	2–57.8	27–274	0.02–0.74	10.46–27.93
		(*n* = 36)	(*n* = 30)	(*n* = 35)	(*n* = 35)	(*n* = 34)	(*n* = 24)
Pubescent	3	207 (± 6)	9.5	209 (± 74)	373.5 (± 55.5)	0.5 (± 0.1)	57.6 (± 4.9)
		201–220	(± 0.5)	135–283	318–429	0.42–0.66	52.68–62.45
		(*n* = 3)	9–10	(*n* = 2)	(*n* = 2)	(*n* = 2)	(*n* = 2)
Mature	24	215 (± 3)	14.4 (± 1.0)	1921.2 (± 294.4)	666.8 (± 34.2)	2.7 (± 0.4)	92.0 (± 8.2)
		190–241	7.5–26	492–5796.5	275–965	0.88–7.05	51.13–165.53
		(*n* = 23)	(*n* = 21)	(*n* = 24)	(*n* = 23)	(*n* = 23)	(*n* = 19)
Total	64	1734 (± 5) 89—241 (*n* = 63)	7.7 (± 0.9) 0–26 (*n* = 53)	771.5 (± 165.5) 2–5796.5 (*n* = 61)	351.8 (± 35.6) 27–965 (*n* = 60)	1.1 (± 0.2) 0.02–7.05 (*n* = 59)	50.6 (± 6.5) 10.46–165.53 (*n* = 45)

**Fig. 2:**
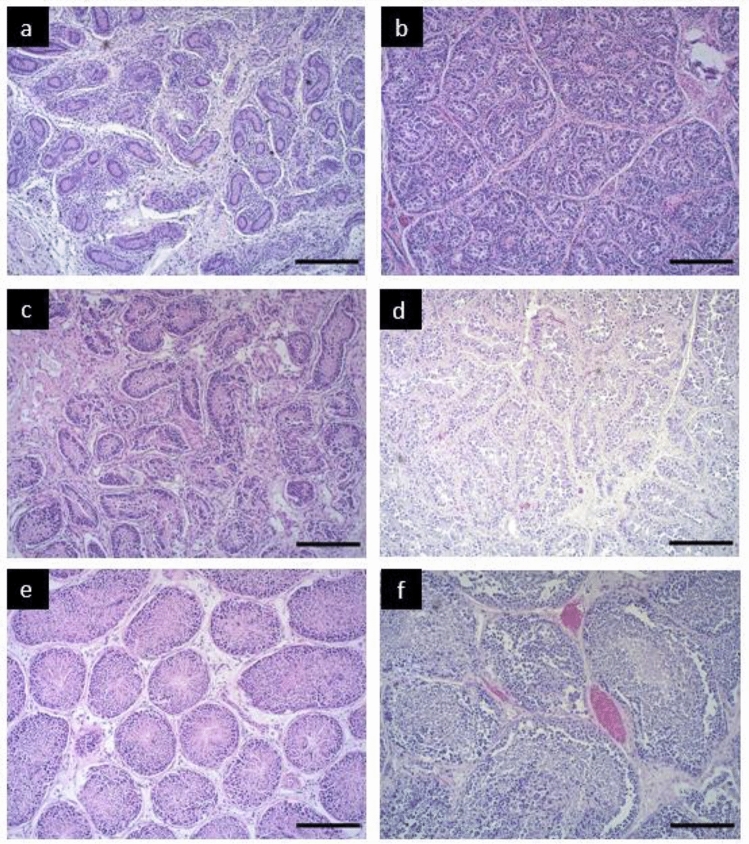
Histological appearance of immature, pubescent, and mature common dolphin (*Delphinus delphis*) testes examined from New Zealand waters (1999–2020). Shown are two individuals for each maturity stage and the scale bar is 100 µm. **a** KS14-63Dd; immature, TBL 95 cm, combined testes weight 3.1 g, mean seminiferous tubule diameter 18.95 µm, and **b** KS20-30Dd; immature, TBL 163 cm, combined testes weight 10 g, mean seminiferous tubule diameter 27.93 µm. **c** KS19-12Dd; pubescent, TBL 201 cm, combined testes weight 429 g, mean seminiferous tubule diameter 52.68 µm, and **d** KS10-78Dd; pubescent, TBL 201 cm, mean seminiferous tubule diameter 62.45 µm. **e** KS20-05Dd; adult, mature, TBL 210 cm, combined testes weight 1505 g, mean seminiferous tubule diameter 109.18 µm, and **f** KS17-01Dd; adult, mature, TBL 208 cm, combined testes weight 2430 g, mean seminiferous tubule diameter 165.53 µm

From the sample set, 18 individuals had missing data and/or testicular tissue too autolysed to assess testicular features such as seminiferous tubule measurements. Histological appearances of immature, pubescent, and mature testis and epididymis are shown in Fig. 2 and described herein.

Immature testes (Fig. 2a, b, n = 37) had seminiferous tubules that were narrow (*x̄* = 17.3 ± 0.9 µm; range 10.5–27.9), tightly arranged, and embedded in abundant interstitial tissue. Enclosed by the basement membrane were one or two layers of two types of cells: the supportive Sertoli cells, and spermatogonia (germinal cells). These cells were undergoing mitosis and neatly aligned the edges of the tubules. The epididymis was empty and exhibited a resting epithelium, indicating that it was undeveloped. In immature testes (and epididymides), no spermatocytes, spermatids, or spermatozoa were observed.

Pubescent testes (Fig. 2c, d, n = 3) contained medium-sized seminiferous tubules (*x̄* = 57.8 ± 4.9 µm; range 52.7–62.5), with spermatogonia and spermatocytes present. A reduction in interstitial tissue and Sertoli cells was noted. Zonation of the spermatogonia and spermatocytes was evident as spermatogonia were undergoing meiosis to produce spermatocytes. No spermatozoa present in the epididymis.

Mature testes (Fig. 2e, f, n = 24) contained large seminiferous tubules with a mean diameter of 92.0 ± 8.2 µm (range 51.1–165.5). All cell types involved in spermatogenesis were present, with spermatocytes, spermatids, and spermatozoa observed in high proportions. Low proportions of interstitial tissue, Sertoli cells, and spermatogonia were noted. Spermatozoa were present in the lumen of the tubules and the epididymis, and the epididymis was enlarged.

### Models of maturity status given with demographic variables

**Fig. 3:**
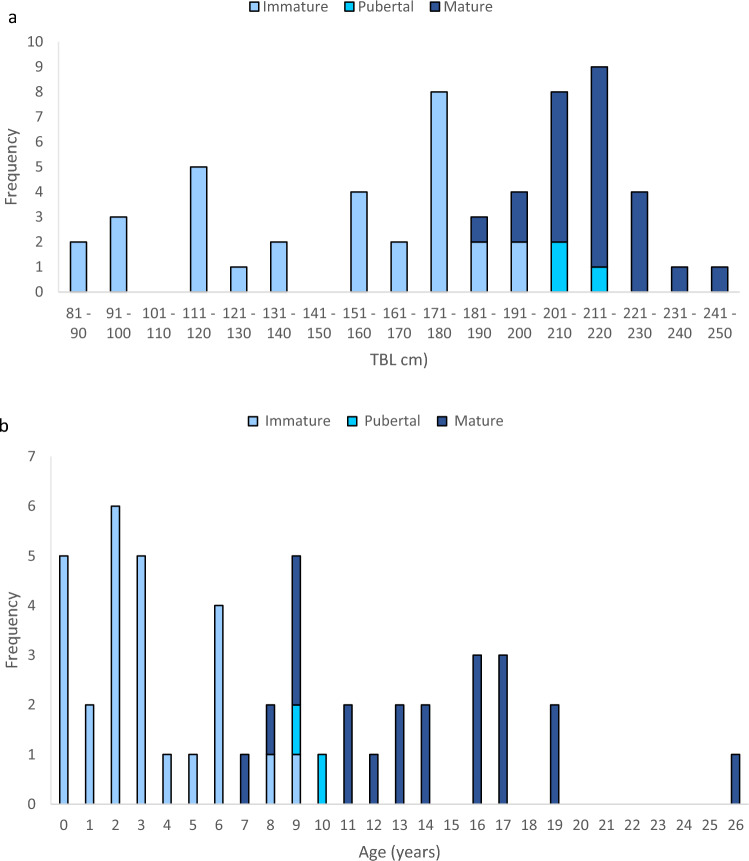
Frequency of distribution of each maturity stage at **a** TBL (*n* = 62) and **b** age (*n* = 55) for male common dolphins stranded and bycaught on the New Zealand coast between 1999 and 2020

Males ranged from 89 to 241 cm in TBL (*n* = 62; two individuals were excluded due to missing TBL data), with a modal size class of 211–220 cm (median = 178 cm; Fig. 3a). The estimated age of male common dolphins ranged from 0 to 26 years (*n* = 55), with two males (KS08-11Dd and KS18-01Dd) only having minimum ages estimated. Of the males that had sexual maturity stage determined, 82% (*n* = 40) of individuals were aged less than 15 years. Immature males ranged from 89 to 200 cm in length and 0 to 9 years in age. Pubescent males ranged from 201 to 220 cm in length and 9 to 10 years in age. Mature males ranged from 190 to 241 cm in length and 7.5 to 26 years in age. Age and TBL increased with maturity stage (Table 1 and Fig. 3), though some overlap between age 8 and 9 years, and 195 cm and 199 cm were evident.

Bayesian modelling estimated the ASM and LSM to be 8.8 years (95% CrI = 7.8–9.8, *n* = 51) and 198.3 cm (95% CrI = 191.4–204.9, *n* = 61; Table 2 and Fig. 5), respectively. Model comparison based on ELPD_LOO scores for the dataset with both age and length (*n* = 34) provided little statistical support for a difference between the utility of age and TBL as indicators of maturity stage, given the difference in ELPD_LOO scores (1.31) was less than the respective standard errors (SE = 3.68 and 3.19). Using the larger ‘age vs TBL’ dataset (*n* = 52), the ELPD_LOO score for the model with age was 2.2 units greater than that of the model using TBL, but the large standard error (SE = 4.1) once again indicated no evidence of one being a better predictor than the other. Applying the SOFI method for comparison across previously studied populations (Ferrero and Walker 1995; Murphy et al. 2005; Westgate and Read 2007), the ASM and LSM were estimated as 8.8 years (SE = 0.16, *n* = 11) and 200 cm (SE = 0.15, *n* = 13), respectively, where *n* is the number of individuals in the indeterminate age or length classes (i.e., age or length classes in which both immature and mature individuals occur in the sample).

**Table 2:** Estimates of the age, total body length (TBL), combined testes weight, combined testes length, index of testicular development (index)m and mean (*x̅*) diameter of seminiferous tubules (mean DT) at the transition values for each case/estimate cutoffs for each model i.e., from immature to pubescent and pubescent to mature, for male common dolphins (*Delphinus delphis*) examined in New Zealand waters between 1999 and 2020

Indicator	*n*	Immature to pubescent $\overline{x }$ (95%CrI)	Pubescent to mature $\overline{x }$ (95%CrI)	ELPD_LOO (SE)
Age (years)	53	7.86 (6.74–8.91)	8.77 (7.81–9.84)	−6.28 (3.19)
TBL (cm)	63	190.80 (183.38–197.81)	198.33 (191.39–204.89)	−4.97 (3.68)
Combined testes weight (g)	61	88.44 (58.45–146.00)	301.62 (156.51–471.20)	−0.31 (0.10)
Combined testes length (mm)	60	279.77 (236.36–334.30)	351.38 (290.50–426.77)	−4.69 (3.15)
Index (g/mm)	59	0.83 (0.76–0.90)	0.92 (0.85–0.98)	−3.72 (3.08)
Mean DT (µm)	45	35.57 (27.54–45.52)	49.97 (40.62–58.26)	−1.08 (0.65)

Posterior distributions of model parameters were obtained using Bayesian cumulative logit regression based on all the data available for each measure in turn; and estimates presented here are summarised with means and 95-percentile credible intervals (CrI) of the posterior distributions of model parameters credible intervals.

*CrI* credible interval; *ELPD_LOO* = Expected Log Predictive Density_Leave-One-Out (all variables together; *n* = 34); *SE* standard error

### Models of maturity status given with testicular variables

**Fig. 4:**
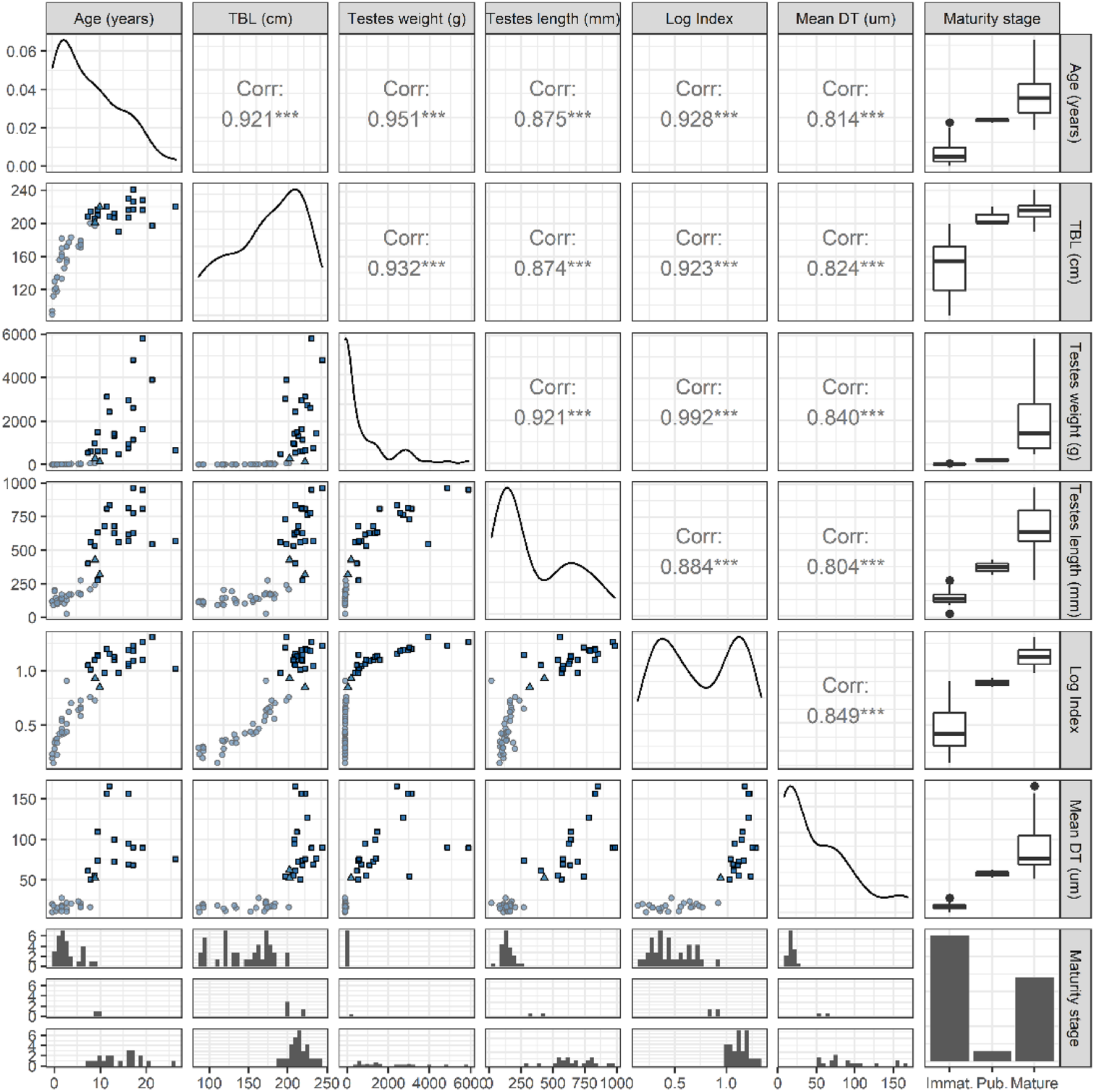
Demographic variables (age and TBL) versus testicular variables (combined testes length [age: *n* = 52, TBL: *n* = 59], combined testes weight [age: *n* = 52, TBL; *n* = 60], log of index of testicular development [age: *n* = 51, TBL: *n = 58]*, and mean diameter of seminiferous tubules* [mean DT; age: n = 37, TBL: n* = 45]) for male common dolphins (*Delphinus delphis*) examined in New Zealand waters (1999–2020)

**Fig. 5:**
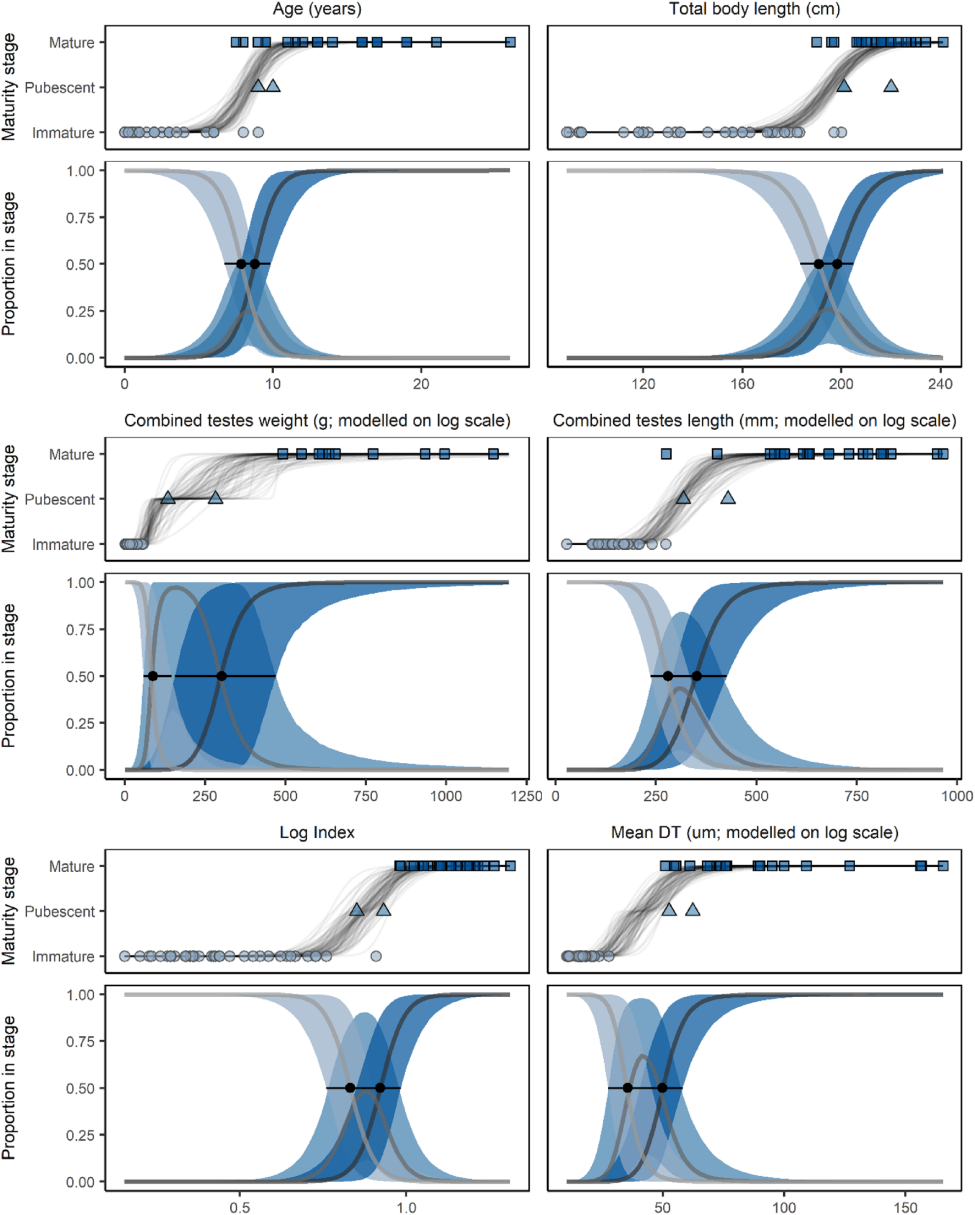
Bayesian cumulative logit regression of the sexual maturation of male common dolphins (*Delphinus delphis*) examined in New Zealand waters (1999–2020) through three stages (immature, pubescent, and mature) modelled as a function of one of six individual measures: age, total body length, combined testes weight, combined testes length, index of testicular development (combined testes weight/combined testes length), and mean seminiferous tubule diameter (mean DT)

Testicular variables (combined testes weight, combined testes length, index of testicular development, and mean seminiferous tubule diameter) for immature, pubescent, and mature male common dolphins are summarised in Table 2. The testicular and demographic variables were all highly correlated with each other (Fig. 5). A general pattern of increasing size with maturity stages was observed with testicular variables, though some overlap between the stages was evident (Table 1, Fig. 4). A significant difference (*p* < 0.001) between the combined testes weight of immature (*x̄* ± SE: 15.3 ± 2.6 g) versus mature (*x̄* ± SE: 1921.2 ± 294.4 g) individuals was detected. A similar difference (*p* < 0.001) was further observed in the mean seminiferous tubule diameter between immature (*x̄* ± SE: 17.3 ± 0.9 µm) and mature (*x̄* ± SE: 92.0 ± 8.2 µm) males.

Combined testes weight and length were both larger in sexually mature animals (8.8 years of age, 198.3 cm TBL). However, significant variation in combined testes weight (492–5796 g) and combined testes length (275–965 mm) remained evident in mature males (Fig. 4). A steep increase in the mean seminiferous tubule diameter was further observed at approximately 190–200 cm TBL and 8–10 years of age (Fig. 4). An estimated 50% of males had reached sexual maturity at 351.38 mm combined testes length, 301.62 g combined testes weight, 0.92 index of testicular development, and 49.97 µm seminiferous tubule diameter (Table 2, Fig. 5). Comparison of the modelled variables with ELPD_LOO indicated that combined testes weight (g) was the best indicator of sexual maturity (Table 2). All testicular variables were better predictors of sexual maturity than age or TBL.

Note that all variables, demographic and testicular, were highly positively correlated (Spearman’s rank correlation coefficients). The type and colour of the data points represent the individuals’ sexual maturity stage: immature = light blue circle, pubescent = medium blue triangle, and mature = dark blue square. The index of testicular development was log-transformed as it was a better predictor in that form.

Each measure has two plots shown (Fig. 5). The upper plot shows each maturity stage and the data values for the measure within each stage. The lines on the plot represent the posterior predictions of the transitions through stages. The lower plot shows the probability of being in each of the three stages where an increase in maturity stage is shown by lighter to darker lines and intervals, which track left to right. The thick lines show the estimated mean and the 95% credible intervals. The black points show the mean, and the thin horizontal lines show the 95% credible intervals of the estimated value of *x*. This is the point at which 50% of males were classified as pubescent (left point and line) and mature (right point and line). The measures for age and TBL on the *x*-axis are shown on the raw scale, but the models were fitted to log-transformed testicular variables, as indicated.

### Reproductive seasonality

**Fig. 6:**
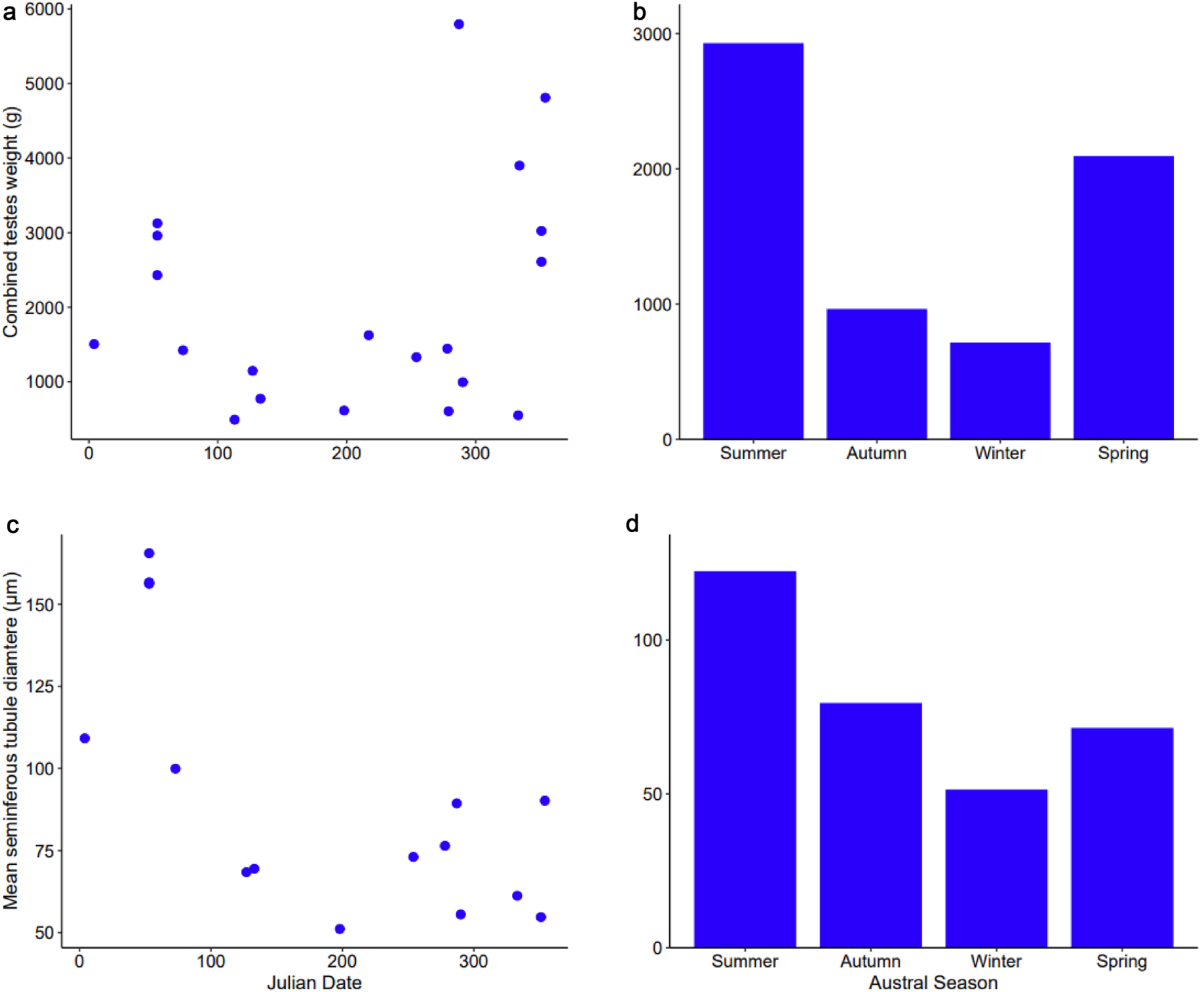
Annual variation in **a** combined testes weight (g) vs. Julian date (*n* = 20) and **c** mean seminiferous tubule diameter (µm) vs. Julian date (*n* = 15) and seasonal variation in **b** combined testes weight (g, *n* = 20) and **d** mean seminiferous tubule diameter (µm, *n* = 15) of mature male common dolphins (*Delphinus delphis*) examined in New Zealand waters between 1990 and 2020

Mean combined testes weight (g) differed among seasons, indicating reproductive seasonality (Kruskal–Wallis test, *p* ≤ 0.01). The greatest and smallest combined testes weights were recorded in austral summer (December–February, *x̄* ± SE: 8923 ± 378 g), and winter (June–August, *x̄* ± SE: 710 ± 505 g; Fig. 6b), respectively. This aligns with the higher combined testes weights recorded at the start (Julian date 0–50) and end (Julian date 300–365) of the year (Fig. 6a). Significant differences in combined testes weight between seasons (*p* = 0.025) were observed. However, no difference in mean tubule diameter was noted between seasons (Kruskal–Wallis test, *p* = 0.106, Fig. 6c and Fig. 6d).

## Discussion

### Stages of sexual maturation

This study examined the reproductive biology of 64 male common dolphins, including both stranded and bycaught individuals. This allowed comparison with prior international studies of male reproduction in common dolphin populations in both the eastern (*n* = 212, Murphy et al. 2005) and western (*n* = 161, Westgate and Read 2007) North Atlantic, and the western South Atlantic (*n* = 54, Grandi et al. 2022). Only three male common dolphins examined in our study were classified as pubescent, with just one having all demographic and testicular variables required for inclusion in Bayesian modelling.

### Comparison of predictors of sexual maturity

For common dolphins, the sexual maturity status, assessed via histological examination, was strongly related to both the demographic variables (age and TBL length) and testicular measurements. In particular, sexual maturity is characterised by a rapid increase in the size of the testis and seminiferous tubules (Murphy et al. 2005; Westgate and Read 2007; this study). This has been observed in other delphinid species such as bottlenose dolphins (*Tursiops aduncus* and *Tursiops truncatus*; Kasuya et al. 1997; Kemper et al. 2014) and pilot whales (*Globicephala melas edwardii*; Betty et al. 2019). The best predictor of sexual maturity in New Zealand common dolphins was combined testes weight, which aligns with international studies (Murphy et al. 2005; Westgate and Read 2007). However, there was substantial overlap in the testicular and demographic variables among the maturity stages, likely due to variation in the maturation process among individuals (Murphy et al. 2005; Kemper et al. 2014; Betty et al. 2019).

The demographic variables, age and TBL, were useful predictors of sexual maturity, although they were not as accurate as the testicular variables (Fig. 5). This has important management implications, as TBL is the easiest and most accessible parameter to collect in the field. Therefore, when post-mortem examinations cannot be undertaken, precise linear measurements of TBL are useful. Additionally, the usefulness of both demographic and testicular variables may change with a larger dataset.

Several challenges come with assessing sexual maturity via histological examination of testicular tissue. For example, testicular tissue of odontocetes is susceptible to rapid post-mortem autolysis (Laws 1961; Kemper et al. 2014; Betty et al. 2019) and can be further impacted by freeze–thaw artefacts which are known to affect histological quality (Schäfer and Kaufmann 1999). In the present study, the testes of some dolphins (*n* = 22) were frozen prior to fixation, while others were fixed fresh (*n* = 23). Such challenges can be mitigated by the exploration of alternative methods to assess sexual maturity in males. For example, blubber testosterone methods, from biopsy samples, can be assessed in live, free-ranging animals and takes away the need to rely on testicular tissue (Kellar et al. 2009). Like any method of data collection, there are still advantages and disadvantages to these alternative methods. Other distinguishing features, such as the prominent post-anal hump, have been used real time (in field) or retrospectively (via photo-ID) to identify mature male common dolphins (Heyning and Perrin 1994; Neumann et al. 2002; Murphy 2004; Ngqulana et al. 2017).

### Attainment of sexual maturity

Male common dolphins in New Zealand waters attain sexual maturity at an average body length (TBL) of 198.3 cm (*n* = 61) as estimated by the logistic regression method (or 200 cm, *n* = 11, as estimated using the SOFI method). These estimates are comparable to previous studies of male common dolphins (Table S2). In the eastern North Atlantic, sexual maturity is attained between approximately 200 (Collet and Saint-Girons 1984; Murphy et al. 2005) and 204 cm (Read et al. 2019). This also aligns with the eastern tropical Pacific where males are estimated to attain sexual maturity at approximately 202 cm (Oliver 1973; Gurevich and Stewart 1978). In the western North Atlantic, males are reported to attain sexual maturity at c.215 cm (Westgate 2005). In contrast, males from the North Pacific attain sexual maturity at c.182 cm (Ferrero and Walker 1995). This likely reflects males in the two populations having different asymptotic lengths (i.e., length at physical maturity). Males in the North Pacific attain physical maturity at 188 cm, whereas males in the western North Atlantic attain physical maturity at 221.5 cm. Asymptotic length is positively correlated with length at sexual maturity, meaning the larger the asymptotic length, the greater the total body length at sexual maturity (Stamps et al. 1998). In the western North Atlantic, immature and mature males overlap between 184 and 209 cm (Grandi et al. 2022).

The average age at attainment of sexual maturity in New Zealand common dolphins is 8.77 years (*n* = 51) as estimated by the logistic regression method (or 8.75 years, *n* = 13, as estimated using the SOFI method). This is the youngest reported ASM estimate for male common dolphins in any international population (Table S2). In the eastern North Atlantic (Irish and French dataset), males attain sexual maturity at 11.86 years (Murphy et al. 2005), and at approximately 10.5 years in North-West Spain (Read et al. 2019) and the North Pacific (Ferrero and Walker 1995). In the western North Atlantic, sexual maturity is attained at 9.45 years (Westgate and Read 2007). In Argentine waters, immature and mature males overlap in age between 6 and 9 years (Grandi et al. 2022). Due to a small sample size (*n* = 52), LSM and ASM estimates were not obtained for the western South Atlantic.

Geographic variation in attainment of sexual maturity can arise from differences in biological and/or environmental factors, including habitat, diet composition, anthropogenic impacts, and population size and density (Murphy 2004; Clutton-Brock and Sheldon 2010; Kemper et al. 2014; Alberts 2019; Barceló et al. 2022). Interestingly, male common dolphins in New Zealand attain sexual maturity at a similar TBL to other populations, despite being at the younger end of reported ASM in international populations. Asymptotic length of males from New Zealand waters (212.1 cm, Palmer 2023), align with asymptotic lengths for males in the eastern North Atlantic (211.6–214 cm; Murphy et al. 2005) and western South Atlantic (211 cm; Grandi et al. 2022). Additionally, New Zealand waters are relatively productive (Murphy et al. 2001), potentially providing abundant prey to support faster growth rates, assuming no disruption to the food web (Stockin et al. 2022, 2023). There is no evidence, however, that male common dolphins in New Zealand have an expedited growth rate (Palmer 2023) compared to other populations (Westgate 2005; Murphy et al. 2005).

Intra-specific competition for access to females may reflect the slightly older age at sexual maturity for males in comparison to females in New Zealand (Palmer et al. 2022). This longer stage of growth before sexual maturity could indicate sexual dimorphism within the population, as a larger size is more favourable for competition (Read et al. 1993; Murphy et al. 2005). Additionally, male common dolphins in New Zealand waters obtain sexual maturity at a marginally younger age compared to their international counterparts (Ferrero and Walker 1995; Murphy et al. 2005; Westgate and Read 2007; Read et al. 2019). This may indicate earlier allocation of resources to testicular mass and the post-anal hump (Ngqulana et al. 2017). Such a strategy may reflect the mating system of common dolphins in New Zealand, where females mate with many males, inducing sperm competition (Murphy et al. 2005).

Alternative explanations for the younger ASM are methodological error and a small sample size. While aging cetaceans is not without error (Hohn 1989; Rosas et al. 2003), an underestimated ASM is unlikely in the current study due to strict quality controls and best practice applied (Murphy et al. 2014). Specifically, blind readings were made across multiple experienced readers, with no *apriori* biological knowledge of the individual. The sample size for this study is smaller compared to most other studies on male reproduction in common dolphins (e.g., Murphy et al. 2005), and so, this may impact the outputs. The authors acknowledge the small sample size of this study, which spans more than a quarter of a century, offering a baseline for which mortality effects on the populations reproductive biology can be monitored. With increasing sample size, the aim is to establish whether the reproductive parameters reported here represent a natural baseline for the population and identify any temporal variation should it exist.

### Reproductive seasonality

Reproductive seasonality was evident in New Zealand male common dolphins, with the largest combined testes weight observed in austral summer (2898.5 ± 464.2 g). Combined testes weight was, on average, three times heavier than in austral winter (710 ± 505 g). Fluctuations in combined testes weight across seasons aligned with testicular activity, which was also greatest in austral summer and lowest in austral winter. Similar patterns have been observed in Northern Hemisphere populations. For example, in the western North Atlantic, the mean mass of regressed testes was 802.9 ± 455.5 g, compared to 4049.6 ± 1317.4 g for testes in full production (Westgate and Read 2007). The full production peak in the western North Atlantic was observed in July (boreal summer) and aligned with the female reproductive cycle for that region. Reproductive seasonality is also observed in the eastern North Atlantic common dolphin population. Spermatogenesis occurs throughout the year, with a marked increase in testicular activity and mass from late May to September. A more active period is reported in July and August, which aligns with the female reproductive cycle (Murphy et al. 2005, 2009).

For males to maximise reproductive output, they align their reproductive cycle with females (Pomeroy 2011). Reproduction tends to also be associated with the availability of resources (Clapham 1996; Bronson 2009). Interestingly, seasonality is not so distinctly observed in females in New Zealand waters, which is more similar to common dolphins examined from the eastern tropical Pacific (Danil and Chivers 2007). For female common dolphins in New Zealand waters, breeding occurs year-round with slight peaks between August and November, which corresponds to late austral winter to late austral spring (Palmer et al. 2022). This does not align with testicular mass (which is associated with sperm production) of male common dolphins in New Zealand waters which is greatest in austral summer. Common dolphins in New Zealand waters have been previously reported to copulate outside the ‘mating period’ (Neumann 2001), which has been presumed to be recreational (Murphy et al. 2005). These previous observations could have been successful copulations as female common dolphins do not appear to have a defined mating period. Additionally, testicular activity occurs year-round, so there is the possibility that mating is still occurring successfully, even though the male and female ‘peaks’ do not align in the New Zealand population.

### Male mating strategy

Although male investment in reproductive tissues varies among delphinid species, there is a strong relationship between body mass and testes mass in cetaceans (MacLeod 2010). Delphinids exhibit one of the greatest investments in male reproductive tissues as well as having the largest testes relative to body size of all amniotes (MacLeod and MacLeod 2009). For example, the most investment is noted in dusky dolphins (*Lagenorhynchus obscurus*) where testicular tissue accounts for up to 8% of total body mass (Van Waerebeek and Read 1994). Common dolphins were ranked comparatively high among cetacean species for the relative investment in testicular tissue as they account for 3.2% of the total body mass in the species (Macleod 2010). Such high reproductive investment is also observed in male common dolphins in both the Northern Hemisphere (Murphy et al. 2005; Westgate and Read 2007) and Southern Hemisphere (this study; Plon et al. 2012). For common dolphins in the western North Atlantic, the testes mass of sexually mature individuals is reported to vary between 2.3 and 4.4% of body weight (Westgate and Read 2007) and for males off of South Africa, on average, testes account for 2% of total body weight (Plon et al. 2012). Sperm competition has been suggested as the primary driver of the large reproductive investment in male common dolphins (Dixson and Anderson 2004), but not the only factor (Kenagy and Trombulak 1986; MacLeod 2010). Common dolphins are considered to have a promiscuous mating system which would support this large investment (Kelley et al. 2014; Ngqulana et al. 2017; Vella et al. 2021). Additionally, as post-anal humps are positively correlated with testis size, it is thought they may act as a visual signal via mate choice by females and establishing dominance hierarchies among males (Lewis 1991; Neumann et al. 2002; Murphy 2004; Murphy et al. 2005; Murphy and Rogan 2006). Future research into how post-anal humps are related to mating systems, maturity, and growth is recommended for the New Zealand population.

## Conclusions

This study provides first insights into the reproductive biology of male common dolphins in New Zealand waters. Knowledge of parameters such as ASM, LSM, reproductive seasonality, and predictors of sexual maturity will aid better understanding of the long-term viability of this population and inform future management decisions. Specific information on the Australasian population is timely, since density-dependent changes may already be in play and, without prior baseline data, remain undetected. Reproductive parameters allow for early detection of population-level changes which is integral for effective species management and conservation. While support exists for a single Australasian population management plan due to the genetic structure of New Zealand and Australian common dolphins, ongoing trans-Tasman collaboration is crucial to allow management of Australasian common dolphins to be effective.

### Supplementary Information

Below is the link to the electronic supplementary material.

Supplementary file1 (DOCX 26 KB)

## Data Availability

The datasets generated during and/or analysed during the current study are available from the corresponding author on reasonable request.
