# Supplementary material for: Reproductive biology of male common dolphins (Delphinus delphis) in New Zealand waters
Source: Mar Biol. 2023 Oct 6;170(12):153. doi: 10.1007/s00227-023-04266-5 (PMC10558376; doi:10.1007/s00227-023-04266-5)
Supplement: Supplementary file 1 — Supplementary file1 (DOCX 26 KB) [file 227_2023_4266_MOESM1_ESM.docx]

## **Reproductive biology of male common dolphins (*Delphinus delphis*) in**

## **New Zealand waters**

Emily I Palmer*^1^, Emma L Betty^1^, Sinéad Murphy^2^, Matthew R Perrott^3^, Adam N H Smith^4^, Karen A Stockin*^1^

^1^Cetacean Ecology Research Group, School of Natural Sciences, Massey University, 0745 Auckland, New Zealand

^2^Marine and Freshwater Research Centre, Department of Natural Resources & the Environment, School of Science and Computing, Atlantic Technological University, ATU Galway City, Old Dublin Road, Galway, H91 T8NW, Ireland

^3^School of Veterinary Sciences, Massey University, Palmerston North, New Zealand

^4^School of Mathematical and Computational Sciences, Massey University, 0745 Auckland, New Zealand

***Corresponding authors**: [e.palmer1@massey.ac.nz](mailto:e.palmer1@massey.ac.nz); [k.a.stockin@massey.ac.nz](mailto:k.a.stockin@massey.ac.nz)

## **Supplementary material**

### Equation S1: sum-of-fraction immature method

The sum-of-fracture of immature method for estimating the average age at attainment of sexual maturity (ASM) was used as follows:

*ASM* = $j+\sum_{i=j}^{K} p_{i}x_{i}$

Variance (s^2^) = $\sum\begin{matrix} \underline{(p_{i}q_{i})x_{i}} \\ N_{i}-1 \end{matrix}$

J is the first indeterminate age class, k is the last indeterminate age class, p_i_ is the proportion of immature specimens in age class i, q_i_ is the proportion of mature specimens in age class i (p_i_ + q_i_ = 1), x_i_ is the number of age classes combined to obtain a sample size of >2 in age class i, I_i_ is the number of immature specimens in age class i, M_i_ is the number of mature specimens in age class i, and N_i_ is the number of specimens in age class i (N_i_ = I_i_ + M_i_). Where, if I_i_  ≠ N_i_, p_i_ = Ii / N_i_, and q_i_ = (M_i_)/N_i;_ if I_i_ = Ni, p_i_ = (I_i_ - ½)/N_i_, and q_i_ = (M_i_ + ½)/N_i_, and if M_i_ = N_i_, p_i_ = (I_i_ + ½)/N_i_, and q_i_ = (M_i_ - ½)/N_i._

The average length at attainment of sexual maturity (LSM) was estimated by modifying the SOFI method, using constant length intervals (5 cm) instead of age (after Danil and Chivers, 2007).

LSM =$j+\sum_{i=i_{\min}}^{i_{\max}} p_{i}x_{i}$

Variance (s^2^) = $\sum_{i=i_{\min}}^{i_{\max}} \frac{p_{i}(1-p_{i})x_{i}}{n_{i}-1}$

J is the lower limit of the length class with the smallest mature animal, i_min_ is the length class with the shortest mature animal, i_max_ is the length class with the longest mature animal, p_i_ is the proportion of immature animals in length class i, x_i_ is the proportion of length classes combined in length class i, n_i_ is the total number of animals in the ith length class.

Table S2: Summary of male common dolphin (*Delphinus delphis*) estimates of average age (ASM, in years) and length (LSM, in cm) at attainment of sexual maturity from published studies. Where available, 95% confidence intervals(CI), credible (CrI) intervals or standard error (SE) are also presented.

| **Location** | **Eastern North Atlantic** | **Eastern North Atlantic – North West Spain** | **Western North Atlantic** | **Eastern tropical Pacific** | **North Pacific** | **Western South Atlantic** | **New Zealand** |
| --- | --- | --- | --- | --- | --- | --- | --- |
| **Reference** | (Murphy, 2004; Murphy et al., 2005) | (Read et al. 2019) | (Westgate 2005; Westgate & Read 2007) | (Oliver 1973) | (Ferrero and Walker 1995) | (Grandi et al. 2022) | This study |
| **Source** | Strandings and bycatch | Strandings and bycatch | Strandings and bycatch | Bycatch | Bycatch | Strandings and bycatch | Strandings and bycatch |
| **ASM**  **(95% CI or CrI)** | 11.86  (SE = 0.62) | 10.5 | 9.45  (SE = 0.19) | Not reported | 10.5  (SE = 0.5) | Immature: 0-9  Mature: 6-23 | Regression = 8.77 (7.81 – 9.84)  SOFI = 8.75  (SE = 0.16) |
| ***n* ASM** | Immature = 136  Mature = 38 | Total = 216 | Immature = 57  Mature = 98 | Not reported | Immature = 50  Mature = 21 | Immature = 12  Mature = 40 | Immature = 30  Mature = 21 |
| **LSM**  **(95% CI or CrI)** | ~200 | 204 | 215 | 202 | ~180  Immature = 179  Mature = 182 | Immature: 97.5 - 209  Mature: 184 - 228 | Regression = 198.33 (191.39 – 204.89)  SOFI = 200  (SE = 0.15) |
| ***n* LSM** | Immature = 140  Mature = 45 | Total = 266 | Immature = 57  Mature = 98 | Total = 405 | Immature = 50  Mature = 21 | Immature = 12  Mature = 40 | Immature = 37  Mature = 24 |
| **Method used** | SOFI | Not reported | SOFI | Not reported | SOFI | Not calculated | Bayesian (regression) and SOFI |
